# Chemical Reactions in Living Systems

**DOI:** 10.1002/advs.202303396

**Published:** 2023-09-07

**Authors:** Dominik Schauenburg, Tanja Weil

**Affiliations:** ^1^ Max Planck Institute for Polymer Research Ackermannweg 10 55128 Mainz Germany; ^2^ Institute of Inorganic Chemistry I Ulm University Albert‐Einstein‐Allee 11 89081 Ulm Germany

**Keywords:** bioconjugation, cellular compartments, click‐chemistry, in vivo chemistry, monitoring reactions

## Abstract

The term “in vivo (“in the living”) chemistry” refers to chemical reactions that take place in a complex living system such as cells, tissue, body liquids, or even in an entire organism. In contrast, reactions that occur generally outside living organisms in an artificial environment (e.g., in a test tube) are referred to as in vitro. Over the past decades, significant contributions have been made in this rapidly growing field of in vivo chemistry, but it is still not fully understood, which transformations proceed efficiently without the formation of by‐products or how product formation in such complex environments can be characterized. Potential applications can be imagined that synthesize drug molecules directly within the cell or confer new cellular functions through controlled chemical transformations that will improve the understanding of living systems and develop new therapeutic strategies. The guiding principles of this contribution are twofold: 1) Which chemical reactions can be translated from the laboratory to the living system? 2) Which characterization methods are suitable for studying reactions and structure formation in complex living environments?

## Graphical abstract

Reactions are often carried out in a controlled laboratory environment using purified chemicals, but in recent years, there has been a shift to carrying out reactions directly in living organisms, known as in vivo chemistry. This approach provides a unique opportunity to study and intervene in biological processes or drug release in real time, in their natural environment.

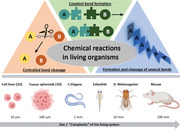

## 1 Introduction – Molecular Complexity in Living Systems

The recent award of the Nobel Prize in Chemistry in 2022, “for the development of click chemistry and bioorthogonal chemistry” to Carolyn Bertozzi, Morten Meldal, and K. Barry Sharpless underlines both the importance and the immense potential of chemical, non‐enzymatic transformations, which may even be performed in systems as complex as living cells, organisms, and animals. However, the history of the development and evaluation of chemical reactions in living systems begins a lot earlier.

More than 200 years ago Friedrich Wöhler discovered that heating inorganic ammonium cyanate resulted in the formation of urea [(NH_2_)_2_CO] – a simple organic compound, which was known to be produced in the kidneys of mammalians.^[^ ^1^, ^2^, ^3^, ^4^ ^]^ It was the first in vitro synthesis of a carbon‐based (organic) chemical and Wöhler`s discovery initiated the downfall of the “*vitalism*” theory, which claimed that organic matter can only be produced in vivo, by living systems. This milestone heralded the birth of organic chemistry and formed the basis for modern drug discovery and medicinal chemistry.^[^ ^1^, ^2^, ^5^, ^6^ ^]^ The biosynthesis of urea, which is much more complex by comparison, was not understood until a century later.^[^ ^5^ ^]^

In organic chemistry, even the most complex chemical reactions can be considered simple in their execution and reaction media, and they mostly allow for the isolation of the product.^[^ ^8^ ^]^ In contrast to this, biological reactions are the chemical processes that occur within living organisms needed to carry out essential functions such as energy production, metabolism, DNA replication, protein synthesis, and enzyme‐catalyzed reactions. They can hardly be surpassed in terms of molecular complexity, selectivity, and efficiency. Biological reactions are highly specific and regulated processes that occur within living organisms, enabling precise transformations and maintaining homeostasis. They are often sensitive to environmental factors and exhibit properties such as reversibility, catalysis, and energy coupling, which contribute to their efficiency and integration within living systems.^[^ ^7^ ^]^ Reactions in living cells have evolved over billions of years. Therefore, they proceed with high chemoselectivity and atom efficiency, which would be hard to achieve by chemical reactions performed in a test tube.^[^ ^9^, ^10^ ^]^ For example, amide bonds are formed in vivo by complex factories called ribosomes yielding polypeptides with distinct amino acid sequences and 3D shapes without the need for protecting groups. The translation of the nucleic acid code into the corresponding protein sequence is accomplished by tRNA macromolecules with precise 3D structures that carry the respective amino acid and recognize nucleotides in the mRNA sequence. This templated amidation reaction proceeds at the site of the unprotected amino acids that are brought in close vicinity by the mRNA. In contrast, synthetic organic chemistry mostly requires sophisticated protection and deprotection reactions to shield reactive groups during synthesis, which adds additional reaction steps, reduces the overall yield, and often requires tedious purification.^[^ ^11^ ^]^

However, we must remember that the elegance and precision we know from biochemical reactions have not always been present. Cellular life is based on nucleic acids (RNA and DNA) and proteins, which have prevailed over time, but it is still an open question as to why and how these essential molecules of life first arose billions of years ago.^[^ ^12^, ^13^, ^14^ ^]^ In 1953, Stanley Miller and Harold Urey postulated that the origin of life could be produced by fairly simple (inorganic) substances.^[^ ^15^ ^]^ In their publication “A production of amino acids under possible primitive earth conditions”, they describe the formation of amino acids in a “primordial soup” after a very short time. These more primitive reactions provide the first insights into how molecular complexity and the building blocks of cellular life have evolved. Nevertheless, it is not yet clear how, for example, the enantioselectivity on which our life is based has emerged.^[^ ^15^ ^]^

Transferring chemical reactions from the laboratory to a living cell or even a multicellular organism seems to be the opposite of what Wöhler postulated two centuries ago. The availability of reactions that allow cleavage and formation of new covalent bonds in vivo enables us to rethink organic chemistry.^[^ ^16^ ^]^

**Figure 1:**
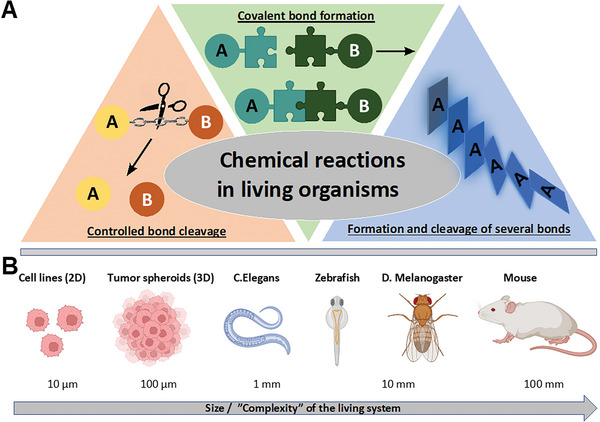
A) Overview of chemical reactions for controlled bond cleavage (left), covalent bond formation (middle), and formation and cleavage of several bonds by, that is, cascade reactions (right) proceeding in living organisms. B) Overview of different living systems from cells to large organisms used for “in vivo chemistry”, with increasing size and complexity.

This could improve our understanding of the sophisticated reaction networks in cells allowing us to learn and predict potential reaction pathways, such as in vivo metabolism of synthetic drug molecules or foods. In addition, we might be able to form entirely new molecules or structures within cells to impart novel functions – an area of great interest for the development of protocells or minimal cells.^[^ ^17^, ^18^ ^]^ 'In the following, we will focus on enzyme‐free chemical transformations of synthetic molecules that are taken up by cells and that occur in cells, or in more complex multicellular living organisms, as shown in **Figure** **1**B. This is in contrast to chemical reactions that naturally proceed within cells, including both enzymatic and non‐enzymatic reactions, which have already been reviewed in detail.^[^ ^19^, ^20^, ^21^, ^22^, ^23^, ^24^ ^]^

We have classified the in vivo reactions into three different categories in this review: 1) controlled cleavage of dynamic covalent bonds, 2) formation of stable covalent bonds, and 3) multistep cascade reactions, involving cleavage and formation of multiple bonds (Figure 1A). Even though reviews exist that summarize these reactions individually,^[^ ^16^, ^25^, ^26^, ^27^, ^28^, ^29^ ^]^ we have gone one step further by comparing these different synthetic strategies, the opportunities, and limitations, we highlight recent developments, and summarize current challenges in monitoring and characterizing reactions within the living system.

## 2 Chemical Reactions in Living Systems

**Figure 2:**
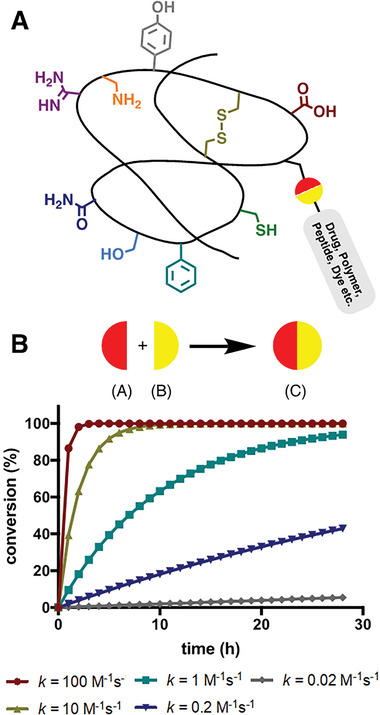
A) Schematic illustration of a chemoselective reaction product. B) Simulation of second‐order reactions between 100 µm reactants (A + B) yielding product (C) as a function of the reaction rate constant. Rate constants of current chemoselective ligation reactions typically range from 10^−3^ to 10^2^ m ^−1^ s^−1^. Reproduced with permission.^[^ ^27^ ^]^ Copyright 2008, American Chemical Society.

Chemical reactions in living systems need to be bioorthogonal, that is, they should proceed in the presence of multiple reactive groups of biomolecules. These transformations are closely related to the field of “click chemistry”,^[^ ^25^ ^]^ which was defined by K. Barry Sharpless as high‐yield, thermodynamically driven chemical transformations, easy to perform, wide in scope, with no or inert by‐products.^[^ ^30^ ^]^ Therefore, a major requirement of these reactions is chemoselectivity.^[^ ^31^ ^]^ A reaction is classified as chemoselective when reagents only react with a particular functional group (like a key in a keyhole), even in the presence of other functionalities (**Figure** **2**A). In the test tube, all “interfering” reactants are usually removed to reduce molecular complexity and often protecting groups are used that direct the reactant to the desired functional group. In living systems, where water, oxygen, small molecules, enzymes, etc., are ubiquitous under dilute conditions of reaction partners in the presence of catalyzing surfaces and interfaces, these strategies could not be effectively applied.^[^ ^17^ ^]^ We should remember that the theoretical considerations of chemical reactions applicable in living systems were mostly measured under very “simple” conditions, for example, the second‐order rate constants (Figure 2B), which reflect the speed of the bimolecular reaction. This means that they were mainly determined in test tubes and in an aqueous buffer, which does not reflect the real in vivo reaction kinetics.

While the term in vivo chemistry describes chemical reactions inside cells or in a complete, living organism, a description of where reactions occur can still be specified.

**Figure 3:**
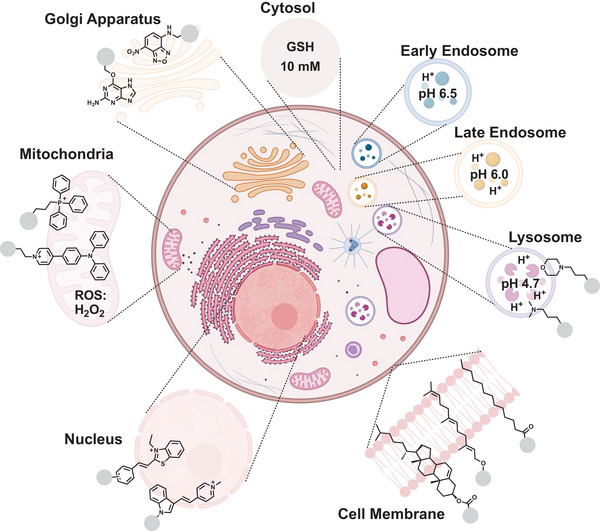
Schematic illustration of compartments in eukaryotic cells, specific chemical triggers of these compartments, and representative examples of small molecule targeting groups.

Eukaryotic cells cannot be seen as a “gray box” where biochemical reactions take place, they are complex factories, which provide different chemical environments in so‐ called subcellular compartments.^[^ ^32^ ^]^ The various organelles in eukaryotic cells include the plasma membrane, mitochondria, lysosomes/endosomes, endoplasmic reticulum (ER), Golgi apparatus, and nucleus, extending from the periphery to the nucleus (**Figure** **3**).

In recent years, considerable work has been done to perform transformations compartment selectively.^[^ ^33^, ^34^, ^35^, ^36^, ^37^, ^38^, ^39^, ^40^ ^]^ Exploiting these interconnected reaction vessels enables us to monitor cell functions, signaling pathways, or stress responses, as well as the selective release or local synthesis of drugs. This can be achieved by (1) selective compartment targeting moieties, or by (2) using a compartment selective chemical “trigger” to facilitate the transformation. Numerous organelle‐targeting fluorescent probes have been developed and extensively reviewed.^[^ ^41^, ^42^ ^]^ In the following, we will summarize small molecule organelle targeting groups, and utilize cellular compartments as reaction vessels, to explain the advances in this fast‐growing field.

### 2.1 Methods to Deliver Molecules to Specific Cellular Compartments

Lipophilic, small molecules (*M*_w_ < 1 kDa) can pass the lipid bilayer of the cell membrane to enter the cytoplasm through passive diffusion.^[^ ^43^, ^44^, ^45^ ^]^ Endocytosis is another mechanism for cellular uptake involving the formation of vesicles that engulf and transport molecules into the cell.^[^ ^46^ ^]^ This process is commonly used for the uptake of larger molecules or particles, such as proteins, lipids, and viruses. Selective delivery to mitochondria can be achieved by low molecular weight, lipophilic and cationic molecules, for example, triphenylphosphonium (TPP) or pyridinium moieties.^[^ ^47^ ^]^ Certain proteins that naturally target the mitochondria, such as cytochrome c, have also been utilized as vehicles.^[^ ^48^, ^49^ ^]^ Acidic organelles (e.g., lysosomes) can be targeted by hydrophobic, weak basic groups. When such a molecule diffuses into the acidic organelle, it becomes protonated and is no longer able to leave.^[^ ^50^ ^]^ For example, chloroquine and its derivatives accumulate in lysosomes and have been used to deliver drugs to these organelles. Antibodies that recognize proteins or lipids on the surface of acidic organelles can be used to selectively deliver drugs or other molecules to these organelles. Just to mention one example, the monoclonal antibody LAMP‐2 targets the lysosome.^[^ ^51^, ^52^ ^]^ Another approach is to use small molecules or drugs that specifically target the Golgi apparatus. In particular, brefeldin A is a drug that disrupts the structure and function of the Golgi apparatus by inhibiting protein transport through the secretory pathway. Other drugs, such as golgicide A, have been developed that specifically target the Golgi, on the other hand, cytotoxicity is observed at higher concentrations, leading to cell death.^[^ ^53^, ^54^ ^]^

The largest cell organelle, the nucleus, can be selectively addressed by small molecules, which can specifically bind to nuclear receptors, altering their activity and thus modulating gene expression, bind to certain DNA sequences or interfere with the activity of DNA‐binding proteins, or scaffolds that modulate epigenetic marks. Besides small molecules, short amino acid sequences (nuclear localization signal peptides such as Lysine‐rich sequences discovered from SV40 Large T‐antigen)^[^ ^55^ ^]^ are recognized by transport proteins in the cell, which then transport the attached cargo into the nucleus, or, for example, lipid‐based nanoparticles can be engineered to specifically target the nucleus of cells.^[^ ^56^, ^57^ ^]^

In general, even though there are many targeting groups that can address specific organelles, the development of chemical reactions that are highly selective in specific organelles, is still in its infancy. This is partly due to the fact that the targeting groups are not as selective as they need to be (at least small molecule targeting groups for different organelles have large structural similarities), given that the bioorthogonal reaction needed for this purpose was only developed in the last 20 years (see chapter 2.3).

Organelle targeting groups can be combined with organelle‐selective cleavage groups so that after transport the drug can be released locally. The underlying chemistry for this “controlled bond cleavage” will be discussed in the next section.

### 2.2 Controlled Bond Cleavage

Various organelles in eukaryotic cells are characterized by a specific local environment that can be used as a so‐called “chemical trigger” (Figure 3). These stimuli, when present in high concentrations, can facilitate chemical transformations in specific compartments. Bond breaking is independent of the initial reactant concentration since the environmental stimulus is usually present in considerable excess (pseudo‐first‐order kinetics).^[^ ^58^ ^]^ In the following section, we will first discuss these reactions. We will then describe the controlled bond cleavage mediated by the addition of another exogenous species, for example, organometallic catalysts.

Reactive oxygen species (ROS), which are mainly produced in mitochondria, can facilitate oxidation reactions, for example, of boronic acids to the corresponding alcohols. In contrast, a high concentration of the cellular reducing agent glutathione (GSH) is present in the cytosol. Here, redox reactions can take place selectively. The physiological pH of the cytosol (7.4) changes dramatically during the endocytosis pathway. The pH of vesicles decreases from 6.5 in the early endosomes to 6.0‐5.5 in the late endosomes, to 4.7 in the lysosome. The high proton concentration can also be taken as a chemical trigger to hydrolyze acetals, hydrazones, or oximes, among others.

**Figure 4:**
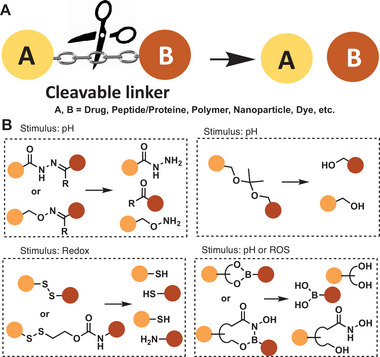
Concept of cleavable linkers by chemical triggers: A) Schematic overview of linker cleavage for selective release. B) Imine‐linkages, hydrazones and oximes, ketals, disulfides, boronate esters, and salicylhydroxamates.

There has been considerable interest in controlling the breaking of (dynamic) covalent bonds by cellular stimuli to release drug molecules from a carrier or by converting a prodrug into an active drug in vivo (**Figure** **4**A). Some of these controlled reactions go back decades. They were often not dubbed “compartment selective” in the initial publications, even though they conform to our current point of view. The strategy of using covalent, but cleavable linkers has been applied to several chemotherapeutic agents that exhibit high cellular toxicity in order to reduce side effects.^[^ ^31^, ^59^ ^]^ The cleavable covalent linkage reacts with cellular or biological stimuli such as acidic pH, reactive oxygen species, or glutathione and the drug becomes active upon unmasking.^[^ ^60^, ^61^, ^62^ ^]^ In the past decade, the U.S. Food and Drug Administration (FDA) has approved more than 30 prodrugs that function via this general mechanism, and the underlying chemistry seems to proceed well in vivo.^[^ ^63^, ^64^ ^]^

**Figure 5:**
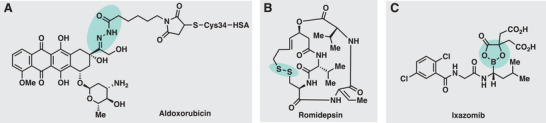
Selected examples of prodrugs that are cleaved by chemical triggers (reactions) in cells.^[^ ^63^ ^]^

Carbonyl moieties (aldehydes and ketones) condensate to hydrazides or hydroxylamines to form hydrazones^[^ ^65^ ^]^ and oximes,^[^ ^66^ ^]^ respectively (Figure 4B). While suitably stable under physiological pH levels, extracellularly or for example in the cytosol, hydrolysis results in controlled bond cleavage under slightly acidic conditions, for example, in the tumor microenvironment or the more acidic intracellular endosomal or lysosomal compartments.^[^ ^65^ ^]^ The ketone in the drug Doxorubicin^[^ ^67^ ^]^ has been used for bioconjugation to Cys34 of the blood plasma protein human serum albumin^[^ ^68^, ^69^, ^70^, ^71^, ^72^ ^]^ by a newly formed hydrazone linker (**Figure** **5**A). This prodrug termed Aldoxorubicin has reached phase III of clinical trials for the treatment of soft tissue sarcoma.^[^ ^73^ ^]^ Furthermore, many cancer cell lines are characterized by higher intracellular glutathione (GSH/GSSG) concentration, allowing the release of redox‐active moieties, for example, from disulfides^[^ ^63^ ^]^ or thioethers in the cytosol.^[^ ^74^ ^]^ The bis‐cyclic, disulfide‐containing drug Romidepsin^[^ ^63^ ^]^ (Figure 5B) was approved by the FDA in 2009. Disulfide reduction by glutathione as a chemical trigger results in a monocyclic dithiol, an active anticancer agent used in cutaneous T‐cell lymphoma, while the disulfide form is inactive.^[^ ^75^, ^76^, ^77^, ^78^ ^]^ Boronic acids, which condense with diols or salicylhydroxamates, have been applied for controlled drug release due to the pH‐induced cleavage of the boronic ester in endosomal or lysosomal compartments.^[^ ^79^, ^80^ ^]^ Ixazomib citrate^[^ ^81^, ^82^ ^]^ bearing a single boronic ester group was approved by the FDA in 2015 for the treatment of multiple myeloma (Figure 5C). The citrate ester stabilizes the oxidation‐sensitive boronic acid.^[^ ^81^ ^]^ Moreover, alkyl and aryl boronic acids and esters can be oxidized to the corresponding alcohols by ROS in the necrotic core of cancerous tissue or the cytoplasm.^[^ ^62^ ^]^ Besides these three representative examples, numerous prodrugs have been reported that could be activated by a chemical trigger in vivo and that have been presented in literature or even received approval by the FDA.^[^ ^64^ ^]^

A selection of dynamic covalent reactions that have been applied as prodrugs, and the required chemical triggers for selective uncaging, are summarized in **Table** **1**.

**Table 1:** Controlled bond cleavage for application in living organisms.^[^ ^63^ ^]^

Conjugate	Released Molecule 1	Released Molecule 2	Chemical trigger
Oxime^[^ ^27^, ^66^ ^]^	O‐substituted hydroxylamine	Aldehyde/Ketone	*pH (acidic)*
Imine^[^ ^16^ ^]^	Amine	Aldehyde/Ketone	*pH (acidic)*
(Acyl)hydrazone^[^ ^27^, ^65^ ^]^	Hydrazide	Aldehyde/Ketone	*pH (acidic)*
Nitrone^[^ ^16^ ^]^	N‐substituted hydroxylamine	Aldehyde/Ketone	*pH (acidic)*
(Hemi)acetal/(Hemi)ketal^[^ ^16^ ^]^	Alcohol	Aldehyde/Ketone	*pH (acidic)*
(Hemi)thioacetal/(Hemi)thioketal^[^ ^68^ ^]^	Thiol	Aldehyde/Ketone	*pH (acidic) or GSH*
Thioester^[^ ^16^, ^83^ ^]^	Thiol	Carboxylic acid	*pH (basic) or nucleophiles (*e.g.*, thiols)*
Thioether^[^ ^74^, ^84^ ^]^	Thiol	GSH‐Michael adduct	*GSH*
Disulfide^[^ ^61^, ^85^, ^86^ ^]^	Thiol	Thiol	*GSH*
Diselenide^[^ ^68^ ^]^	Selenide	Selenide	*GSH or ROS*
Thiol‐Michael conjugate^[^ ^85^, ^87^ ^]^	Thiol	Michael acceptor	*GSH*
Boronate ester^[^ ^68^ ^]^	Diol	Boronic acid/(alcohol)	*pH (acidic) or (Oxidation by ROS)*
Boronate salicylhydroxamate^[^ ^79^, ^80^ ^]^	Salicylhydroxamate	Boronic acid/(alcohol)	*pH (acidic) or (Oxidation by ROS)*
*cis*‐Aconityl moiety^[^ ^68^ ^]^	Amine	Maleimide moiety	*pH (acidic)*

Moreover, esters, amides, carbamates, and phosph(on)ate linkages have been introduced in prodrugs as well.^[^ ^63^ ^]^ However, these groups usually require enzymes to be converted to the active drug and are therefore not covered in this review. While numerous dynamic covalent linkers have been used in drug delivery systems, current and future scaffolds aim to synergistically combine different dynamic covalent groups that react with more than one stimulus at the tumor site, minimizing side effects, such as off‐target cleavage, and the drug release.^[^ ^88^, ^89^, ^90^, ^91^, ^92^ ^]^

In addition to exploiting the cellular stimuli, there are examples where linkers are cleaved by the addition of another exogenous molecule. Organometallic complexes are an emerging field for mediating bond‐cleavage reactions to release bioactive agents.^[^ ^93^, ^94^, ^95^, ^96^ ^]^ In particular, the uncaging of protected amines has been exploited for drug activation.^[^ ^97^, ^98^ ^]^ These protective groups or linkers are added to the amine functional groups to enhance stability or prevent premature reactivity of the drug. The release of these protected amines renders the drug active and allows it to interact with its target molecules or exert its therapeutic effects in the cellular environment.

In particular, the allyloxycarbonyl (alloc) group has proved useful for deprotection by palladium catalysts.^[^ ^99^ ^]^ Here the palladium forms a π‐allyl complex, while the carbamate acts as a leaving group in an allylic position.^[^ ^100^ ^]^ In addition to palladium, ruthenium complexes can also be used for protecting group cleavage.^[^ ^97^, ^101^ ^]^ These complexes reveal high chemical stability and can generate reactive oxygen species (ROS) upon irradiation.^[^ ^102^ ^]^ Therefore, they have been used for photodynamic therapy, in combination with ROS‐sensitive linkers. In these systems, the drug molecules were delivered to the tumor cells in an “inactivated” form. Exposure of the tumor cells to red light‐induced cleavage of the Ru‐complexes through the local formation of ROS and release of the active drug molecule.^[^ ^102^ ^]^

A “metal‐free” pathway was described by Neumann et al. in 2016 when they presented “tetrazine‐responsive self‐immolative linkers” for drug‐molecule release.^[^ ^103^ ^]^ In contrast to the well‐known inverse electron demand Diels‐Alder reactions for covalent bond formation (see section 2.3), the authors used a tetrazine‐triggered decaging of *O*‐vinyl ethers. The rapid and selective reaction of the triazine with these dienophiles allowed to restore the alcohol functional groups.

However, these representative examples are bimolecular reactions, where both the “caged molecule” and the catalyst/reagent have to be present in the same cellular compartment, and have similar pharmacokinetics, which makes in vivo applications difficult.

### 2.3 Covalent Bond Formation

**Figure 6:**
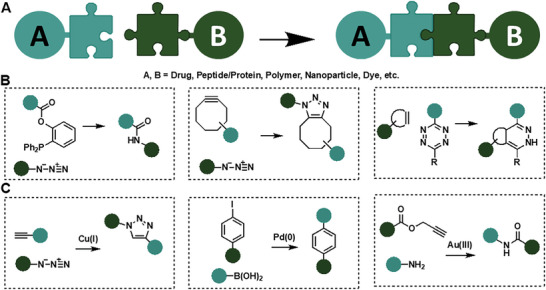
Concept of covalent bioconjugation reactions: A) Schematic overview of covalent bond formation from reactive but also stable functional groups. B) Traceless bioconjugation reactions that do not require any additive or catalyst (Staudinger ligation, strain‐promoted azide‐alkyne cycloaddition (SPAAC), inverse electron‐demand Diels–Alder reaction (IEDDA)). C) Transition metal‐catalyzed bioconjugation reactions (copper(I)‐catalyzed azide‐alkyne cycloaddition (CuAAC), palladium‐catalyzed Suzuki‐Miyaura cross‐coupling, gold‐catalyzed amide bond formation).

The chemoselective formation of covalent bonds between two different molecules plays a key role in every living system. Controlling covalent bond formation in vitro and in vivo is still considered to be the “Holy Grail” for chemists (**Figure** **6**A).

In addition to chemoselectivity, fast reaction kinetics is a characteristic of these reactions. At very dilute nano‐ to micromolar concentrations, typically found in biological systems, little product is formed if the desired reaction proceeds with a slow second‐order rate constant (see Figure 2B).^[^ ^58^ ^]^ Furthermore, the application of chemical reactions in living organisms requires fast kinetics so that bonds can be formed before the reactants are degraded by metabolism or disappear through diffusion.^[^ ^81^, ^104^ ^]^ The significance of this research area was highlighted by the award of the 2022 Nobel Prize in Chemistry to Carolyn Bertozzi, Morten Meldal, and K. Barry Sharpless for the development of “click” and bioorthogonal chemistry.

Several bioorthogonal reactions that proceed in aqueous media with fast reaction kinetics have been identified and are summarized in Figure 6B.^[^ ^26^, ^27^ ^]^ Here, endogenous functional groups react with each other. Therefore, these reactions themselves are independent of the cell compartments, even if organelle‐selective targeting groups are often used.

Carolyn Bertozzi has introduced the well‐known reduction of organoazides by phosphines, termed Staudinger reaction.^[^ ^16^, ^105^, ^106^ ^]^ The aza‐ylide intermediate can be trapped by an acyl‐donor, resulting in amide bond formation upon hydrolysis.^[^ ^16^ ^]^ The first elucidation of cell surface re‐engineering applying azido reporters, a traceless form of the Staudinger ligation, was performed by this group in 2000.^[^ ^106^, ^107^ ^]^ Upon amide bond formation, the linker is eliminated, yielding the amide and triphenylphosphine oxide. This ligation was the first synthetic chemical reaction performed in living cells as azides and phosphines are absent in biological systems, which makes this reaction highly chemoselective. A milestone was reached when Bertozzi and collaborators succeeded in moving from amide‐forming Staudinger ligation in cell lysates or on living cell surfaces to the first reported bioorthogonal reaction in living mice.^[^ ^108^ ^]^

Since the early 2000s, different applications of the Staudinger ligation in living systems have been demonstrated, focusing mainly on the injection of azide‐functionalized glycans into mice for the profiling of glycoproteins.^[^ ^107^ ^]^ A limitation of this efficient method is the stability of phosphine reagents that are nontoxic but become rapidly oxidized in biological systems. Furthermore, the relatively slow reaction kinetics (10^−3^ m ^−1^ s^−1^) limit their applicability.^[^ ^109^, ^110^ ^]^

Pericyclic reactions are a class of organic transformation that involves concerted, cyclic redistribution of electrons. These reactions typically occur in solution and are commonly studied in organic chemistry. However, in the last two decades, pericyclic reactions have been identified which can occur in vitro and in vivo in the same way that they occur in the laboratory, even in the presence of the highly complex and dynamic nature of biological systems. Generally, these reactions are independent of cell compartment selective triggers, nonetheless, selective targeting groups are often utilized.

Sharpless and Meldal identified independently that the 1,3‐dipolar cycloaddition between an organoazide as dipole and an alkyne as dipolarophile, which was reported in the early 1960s by Huisgen,^[^ ^111^, ^112^ ^]^ can be modified for biochemical applications. For a long time, this reaction was disregarded in bio‐organic chemistry, due to its slow reaction kinetic and the formation of two regioisomers. More than 40 years after its discovery, Sharpless and Meldal reported in 2002 a copper(I)‐catalyzed version of Huisgen's 1,3‐dipolar cycloaddition.^[^ ^113^, ^114^ ^]^ Utilizing this ligation, cell surface remodeling, and metabolic labeling have been achieved.^[^ ^16^, ^115^, ^116^ ^]^ In 2003, Cravatt et al. reported enzyme labeling with an azido‐modified protein, followed by a Cu‐click reaction to rhodamine alkyne.^[^ ^117^ ^]^ The reaction occurred in living cells and in a mouse model, where the modified proteins were isolated after in vitro labeling in complex proteomes by click chemistry ex‐vivo.^[^ ^16^, ^114^, ^118^ ^]^ In contrast to the Staudinger ligation, CuAAC proceeds with fast second‐order rate constants (10^0^–10^3^ m ^−1^ s^−1^), but it still requires Cu(I) as a catalyst.^[^ ^119^ ^]^ The toxicity of the transition metal arising is often a limitation of CuAAC, through the binding of Cu(I) to thiols (e.g., GSH or cysteine sidechains in proteins), thereby inactivating the catalyst and triggering oxidative stress.^[^ ^120^ ^]^

**Figure 7:**
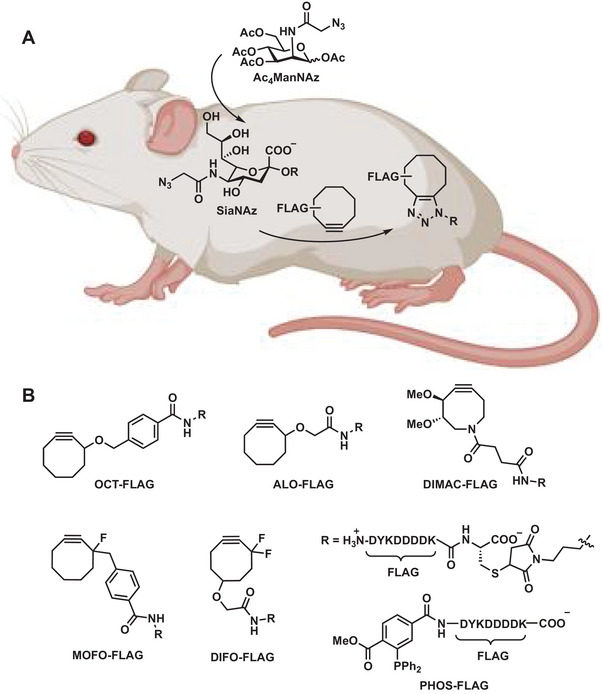
Strain‐promoted click chemistry in mice. A) Mice were injected with *N*‐azidoacetylmannosamine‐tetraacylated (Ac4ManNAz) to allow for metabolic labeling of glycans with SiaNAz. They were then injected with a cyclooctyne‐FLAG conjugate for in vivo covalent labeling of azido glycans. B) Different FLAG conjugates used. Reproduced with permission.^[^ ^119^ ^]^ Copyright 2010, The Authors, published by National Academy of Sciences.

Wu and co‐workers used a zebrafish embryo vertebrate model to perform a click reaction.^[^ ^121^ ^]^ In vivo imaging of fucosylated glycans during early zebrafish embryogenesis was facilitated by glycan alkyne clicked to Alexa Fluor 488‐azide by CuAAC without any toxicity. To overcome the limitations of Cu(I)‐catalyzed Huisgen cycloaddition reactions, Bertozzi and coworkers developed the so‐called strain‐promoted azide‐alkyne cycloaddition reaction (SPAAC) that utilizes cyclooctynes as dipolarophiles (**Figure** **7**).^[^ ^122^, ^123^ ^]^ This reaction was first identified by Wittig and Krebs in 1961.^[^ ^124^ ^]^ Linear alkynes have a bond angle of 180°. In contrast to this, the sp‐hybridized carbon atoms in cyclooctynes form an angle of 160°.^[^ ^16^, ^122^ ^]^ Therefore, these strained alkynes are more reactive toward cycloaddition reactions with organoazides, allowing triazole formation in the absence of Cu(I)‐catalysts.^[^ ^119^, ^125^ ^]^

This reaction has been used for cell surface reengineering. Bertozzi and co‐workers grew zebrafish embryos in the presence of *N*‐azido acetyl galactosamine as a metabolic precursor. These azides reacted with cyclooctynes even in living organisms.^[^ ^125^ ^]^ However, the second order rate constants (10^−1^–10^1^ m ^−1^ s^−1^) are not as fast as Cu(I)‐catalyzed click reactions and the covalent linkage formed is bulkier and more hydrophobic than an amide bond (i.e., formed after a Staudinger ligation) or the small triazole in CuAAC.^[^ ^119^ ^]^ Furthermore, side reaction of the bulky and often hydrophobic cyclooctynes with nucleophiles (e.g., thiols) may limit its applicability.

The inverse electron‐demand Diels–Alder ligation (IEDDA) goes back to a long‐known transformation, namely the Diels–Alder reaction. It utilizes an electron‐poor diene, which reacts with an electron‐rich dienophile in a [4+2] cycloaddition reaction.^[^ ^26^, ^126^ ^]^ This reaction shows considerable rate enhancement in polar solvents making it attractive for applications under physiological conditions.^[^ ^26^ ^]^ The most common IEDDA system used today consists of a tetrazine as a diene and a strained alkene or alkyne as a dienophile. After the first [4+2] cycloaddition of the dienophile with the tetrazine, a bridged intermediate is formed, which then undergoes a retro‐Diels–Alder reaction expelling nitrogen yielding the dihydropyridazine product.^[^ ^126^ ^]^ Second‐order rate constants of tetrazine IEDDA can be tailored by varying the ring strain of the electron‐rich dienophile. Click reactions utilizing *trans*‐cyclooctenes are among the fastest, non‐enzymatic bond formations, which are known today (K_rel_ up to 10^6^ m ^−1^ s^−1^).^[^ ^26^ ^]^

**Figure 8:**
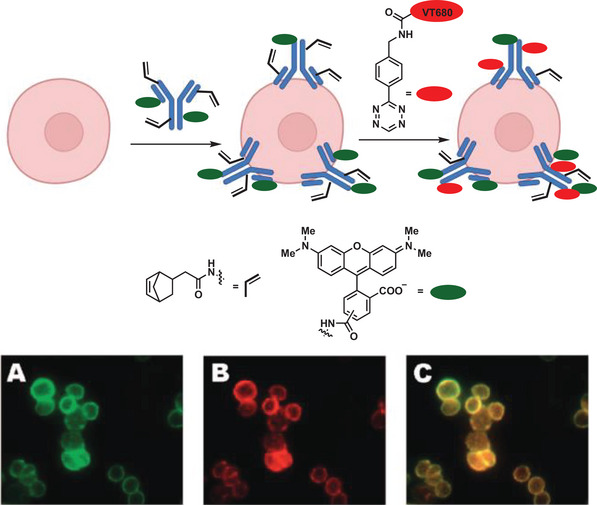
Pretargeting of SKBR3 cells with norbornene and tetramethylrhodamine co‐labeled trastuzumab, followed by tagging the live cells with tetrazine‐VT680. A) Rhodamine channel. B) Near‐IR channel (tetrazine‐VT680). C) Merged image of A and B. Adapted with permission.^[^ ^127^ ^]^ Copyright 2008, American Chemical Society.

The first tetrazine click reaction in living cells was reported by Hildebrand and co‐workers in 2008, utilizing pretargeted norbornenes as dienophiles (**Figure** **8**).^[^ ^127^ ^]^ A monoclonal antibody, which is a highly specific biomarker, modified with norbornene was utilized to target Her2/neu receptors on live human breast cancer cells (pretargeting). An inverse electron demand Diels‐Alder coupling to a near‐infrared fluorophore (tetrazine‐VT680) was performed and rapidly labeled antibodies were observed.

**Figure 9:**
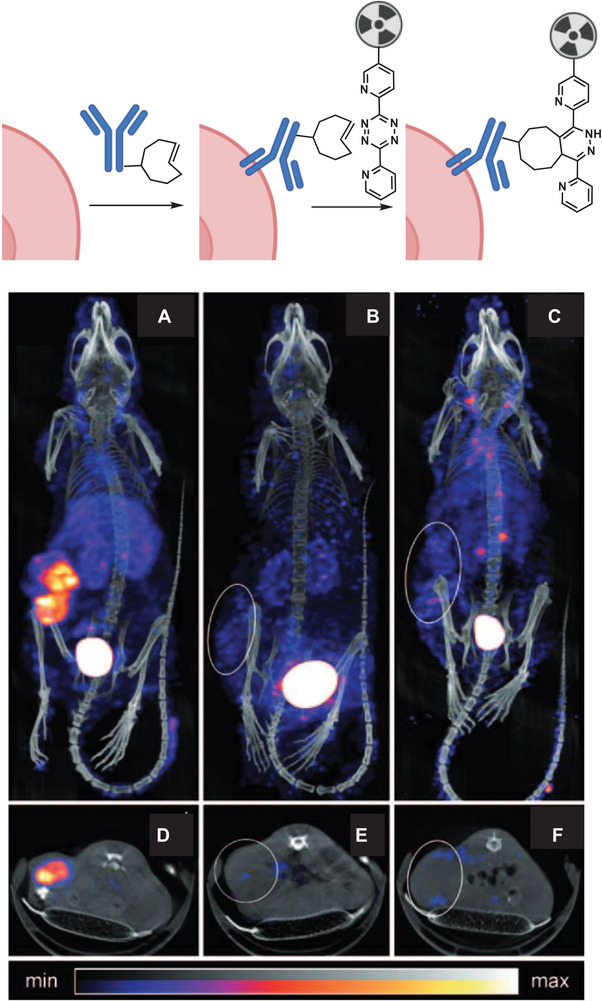
General scheme of tumor pretargeting by using the inverse‐electron‐demand Diels–Alder reaction. Small animal single photon emission computed tomography of live mice bearing coloncarcinoma xenografts. Reproduced with permission.^[^ ^128^ ^]^ Copyright 2010, Wiley‐VCH.

When Robillard et al. reported in 2010 the first example of reacting two exogenous functional groups in a living animal followed by noninvasive imaging using a pretargeting approach (**Figure** **9**),^[^ ^128^ ^]^ a breakthrough in chemical reactions performed in living animals was achieved.

A *trans‐*cyclooctene (TCO) modified monoclonal antibody was injected into mice bearing colon carcinoma and after 24 h, the tetrazine functionalized [^111^In]‐radiolabeled probe was administrated. A blood clearance half‐life of 9.8 min was observed for the [^111^In]‐tetrazine probe, elucidating the importance of fast reaction kinetics. This approach allowed the localization of radioactivity in the living animal and demonstrated selective delivery of the tetrazine to its reaction partner (TCO). In addition to these well‐known click reactions modified for in vitro and in vivo applications, newly developed transition metal‐catalyzed transformations have been used for chemical reactions in living organisms.^[^ ^129^ ^]^ Intracellular biochemical reactions often rely on transition metal catalysts, commonly in the form of metalloproteins.^[^ ^87^ ^]^ Non‐enzymatic transition metal catalyzed reactions are limited in their applicability due to cytotoxicity of the metal and side reactions (catalyst poisoning) with cellular components, for example, glutathione.^[^ ^120^, ^131^, ^132^ ^]^ On the other hand, no covalent bond formation is observed in the absence of a catalyst (low background reaction) leading to high selectivity. Palladium‐catalyzed cross‐coupling reactions are an emerging class of carbon–carbon bond formation reactions.^[^ ^130^ ^]^

**Figure 10:**
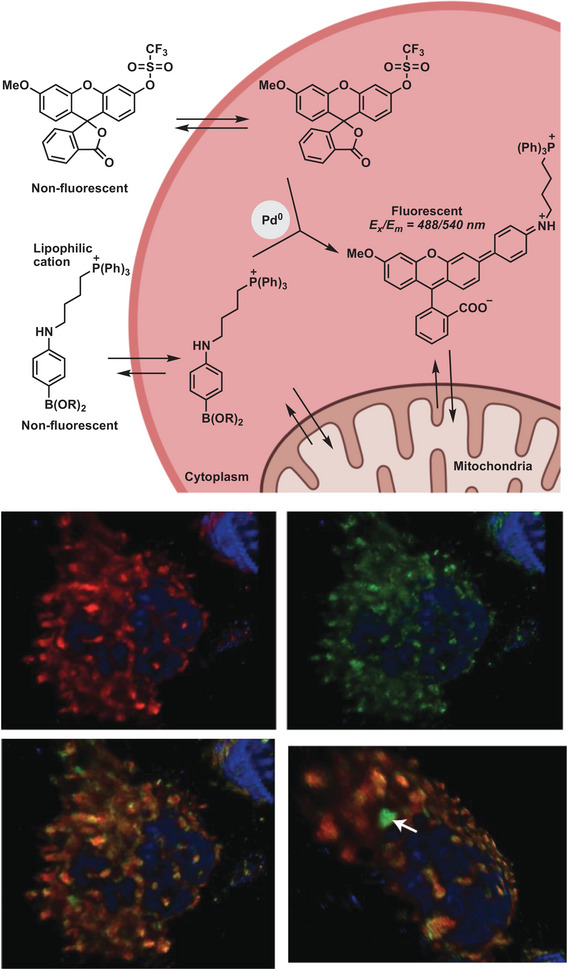
Pd^0^‐mediated Suzuki–Miyaura cross‐coupling within HeLa cells. Pd^0^‐catalyzed intracellular cross‐coupling generates the mitochondria‐localized fluorescent compound. Reproduced with permission.^[^ ^129^ ^]^ Copyright 2011, Springer Nature.

**Figure 11:**
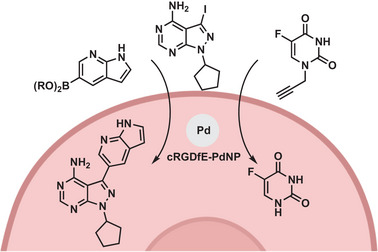
Pd‐catalyzed activation and synthesis of two anticancer agents. Simultaneous decaging of the alkyne and Suzuki–Miyaura cross‐coupling reaction of aryl iodide and aryl boronic acid. Reproduced with permission.^[^ ^135^ ^]^ Copyright 2017, Wiley‐VCH.

High selectivity under physiological conditions along with good functional group tolerance distinguish the Suzuki–Miyaura^[^ ^133^ ^]^ and the copper‐free Sonogashira^[^ ^134^ ^]^ reactions. In 2011 the Bratley group presented different intracellular palladium‐mediated reactions, utilizing Pd^0^ nanoparticles (**Figure** **10**).^[^ ^129^ ^]^ The catalyst is able to cross the cellular membrane and remains in the cytoplasm for days without interfering with cell viability. Intracellular Suzuki–Miyaura cross‐coupling of a non‐fluorescent aryl triflate and boronic acid enabled in vitro synthesis of a new fluorescent dye. The same group proposed six years later the chemical synthesis of two different anti‐cancer agents in glioblastoma cells, mediated by cancer‐targeting palladium catalysts.^[^ ^135^ ^]^ Multi‐targeted kinase inhibitor (PP‐121) was synthesized by the Suzuki–Miyaura reaction in vitro in addition to the activation of fluorouracil prodrug, resulting in increased cell toxicity compared to individual treatments (**Figure** **11**). The intrinsic toxicity observed for copper and nickel cations, limiting, for example, in vivo applications of CuAAc, is not observed for late 4d and 5d transition metals when coordinated in a ligand sphere.^[^ ^129^ ^]^

**Figure 12:**
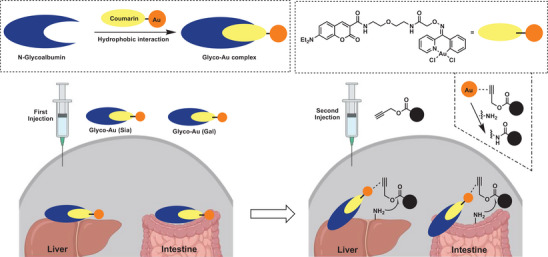
Organ‐selective accumulation within live mice directed by disialo‐ and galactosyl‐linked glycoalbumins. Preparation of glycoalbumins as “transition‐metal carriers” to produce Glyco‐Au complexes. Glyco‐Au (Sia) and Glyco‐Au (Gal) were synthesized with (2‐6)‐disialoglycoalbumin and galactosylglycoalbumin, respectively. General scheme for liver‐ and intestine‐selective in vivo fluorescence labeling by Au(III)‐catalyzed amide bond formation between propargyl ester‐based imaging probes and surface amino groups of targeted tissues. Reproduced with permission.^[^ ^136^ ^]^ Copyright 2017, Wiley‐VCH.

While in the past different powerful approaches for metal‐catalyzed reactions have been demonstrated in biological systems, such as in various mammalian cell lines and bacterial cells, the first transition metal‐catalyzed covalent bond formation in living animals (mice) was demonstrated by Tsubokura et al. in 2017 (**Figure** **12**).^[^ ^136^ ^]^ Albumin was used as a metal complex carrier and targeting vessel. By decorating the albumin surface with different N‐glycan molecules organ‐selective accumulation was achieved (glycan‐targeting). Propargylic esters served as acyl donors after activation with an Au(III) catalyst on glycan‐targeted organs, allowing target‐selective labeling by nucleophilic exchange with amines on surface proteins of the target tissue.

Chemical reactions successfully applied in living organisms are summarized in **Table** **2** in ascending order of their determined second‐order rate constants. A critical evaluation of the positive and negative aspects is given.

**Table 2:** Covalent bond formations by chemical reactions suitable for application in living organisms.^[^ ^129^ ^]^

Reaction	Additive/Catalyst	Second‐order rate constant [m ^−1^ s^−1^]	Application field	Pro	Contra
Staudinger ligation^[^ ^106^, ^107^ ^]^	none	10^−3^	in vivo/in vitro	Native amide bond	Slow reaction kinetics
Native chemical ligation^[^ ^16^, ^83^ ^]^	none	10^−2^–10^−1^		Native amide bond	Thioester is prone to hydrolysis
Gold‐catalyzed amidation^[^ ^136^ ^]^	Gold	n.d.	in vivo/in vitro	Native amide bond	
Olefin metathesis^[^ ^16^ ^]^	Ruthenium	10^−1^	in vitro	Stable carbon‐carbon bond formed	Olefins can serve as Michael‐acceptor
Cross‐coupling^[^ ^16^ ^]^	Palladium	10^−1^–10^0^	in vivo/in vitro	Low background reaction in the absence of Pd	Toxicity of Pd, oxidation of boronic acids
SPAAC^[^ ^122^, ^125^ ^]^	none	10^−2^–10^0^	in vivo/in vitro		Bulky/hydrophobic bond, reaction with thiols
SPANC^[^ ^16^, ^26^ ^]^	none	10^0^–10^1^			Bulky/hydrophobic bonds, nitrones unstable
Photo click^[^ ^26^ ^]^	UV light	10^1^	in vitro		Requires UV light
CuAAC^[^ ^16^, ^113^ ^]^	Copper	10^0^–10^2^	in vitro	Peptide bond surrogate	Toxicity of Cu
IEDDA^[^ ^26^, ^137^ ^]^	non	up to 10^6^	in vivo/in vitro	Very fast on rate	Bulky/hydrophobic bonds

### 2.4 Formation and Cleavage of Several Bonds and Cascade Reactions

In the past, prodrugs were usually released by a single stimulus. However, in recent years combinations of two or more dynamic covalent bonds have been developed that can respond to different stimuli. Peptide linkers containing a combination of boronic esters with fast and disulfide groups with slow association and dissociation rates have thus emerged.^[^ ^138^ ^]^ Such dual‐responsive linkers allowed pre‐coordination through the boronic acid‐catechol interaction, resulting in improved self‐sorting and higher selectivity in the formation of disulfide heterodimers. The resulting bis‐peptide conjugate exhibits improved stability in the acidic tumor‐like extracellular microenvironment. In addition, it responds to pH changes within the physiological range and to redox conditions present in cancer cells, which is promising when controlling the stability of the bioconjugates upon dilution in cellular environments through cooperative effects. We can also envision smart drugs that respond to several different biological stimuli in cells and tissue to improve cell or tissue‐specific delivery.

However, dual responsiveness can also be used in a different way other than to provide higher selectivity. Thioether groups respond to both glutathione and reactive oxygen species as chemical triggers. Wang et al. have used thioethers in docetaxel prodrugs.^[^ ^84^ ^]^ Its release is faster than the “mono‐responsive” linker because it responds to two opposing stimuli.

In addition to these highly selective, dual‐responsive mechanisms, two other general reaction pathways can be distinguished. One is a (supramolecular)polymerization of small molecules in living organisms^[^ ^139^ ^]^ and the other is a de‐polymerization releasing small molecules from a macromolecule in high precession.^[^ ^140^ ^]^

Some organelles, such as nuclear pore complexes and peroxisomes, are formed through the aggregation of specific proteins. However, the formation of organelle‐like structures can be mimicked chemically. One example of supramolecular polymerization in cells is the formation of amyloid fibrils, which are protein aggregates associated with various neurodegenerative diseases.^[^ ^141^ ^]^ Amyloid fibrils are formed through the self‐assembly of proteins, such as amyloid beta, into beta‐sheet‐rich structures that then assemble into fibrils, inspiring the chemist to utilize a similar reaction pathway. Additionally, supramolecular polymers have been used to create synthetic membranes that can mimic the properties of cell membranes.

**Figure 13:**
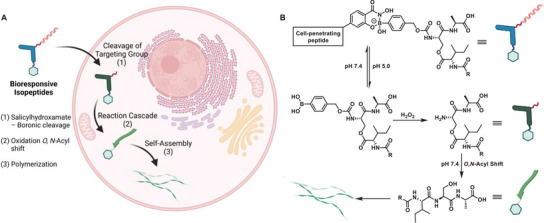
A) Schematic overview of Intracellular co‐assembly of peptides; B) Chemical reactions leading to cellular uptake, peptide linearization, and peptide co‐assembly.^[^ ^80^ ^]^

Recently our group demonstrated that pre‐assembling molecules are transported into cells through targeting peptides attached by the pH‐responsive salicylhydroxamate – boronic acid interaction that is cleaved in the acidic endosomal compartments and oxidized and rearranged in the cytoplasm for peptide nanofibers (**Figure** **13**).^[^ ^80^ ^]^ Furthermore, we showed that metallo‐peptides can undergo step‐wise transformation into the NIR emitting nanofibers that inhibited cell respiration such as aerobic glycolysis and oxidative phosphorylation in cancer cells.^[^ ^142^ ^]^

Once inside the cell, self‐sorting peptides can undergo self‐assembly to form supramolecular structures that target specific organelles.^[^ ^143^, ^144^ ^]^ The assemblies can be designed to be stable or transient, allowing for the controlled release of cargo molecules within the targeted region. Liu et al. have demonstrated that GSH‐induced self‐sorting into isolated nanofibrils can be targeted to the ER and the Golgi apparatus, thus leading to combinatorial organelle dysfunction and death of HeLa cells.^[^ ^143^ ^]^ In addition to the disulfide cleavage presented here, other stimuli for the self‐sorting of peptides have been realized, for example by light or enzymes.^[^ ^144^, ^145^ ^]^

Cascade reactions that form or cleave several bonds based on multiple cellular stimuli are emerging and offer the possibility of creating highly specific reaction networks in cellular environments, which is of great importance for the development of minimal or protocells.

As we are focusing on chemical reactions, multi‐enzymatic cascade reactions, as they often occur in nature, are not considered in this review.^[^ ^146^ ^]^

#### 2.4.1 Polymerization by Covalent Bond Formation

Covalent bond formation between many monomers allows the formation of long chain‐like macromolecules.^[^ ^147^ ^]^ The “classical” olefin polymerization, in which chain growth is generated by a radical species, which is then able to add further monomers, is highly challenging when transferring from the laboratory to living cells due to the many interfering molecules, oxygen, and low concentrations. The foundation for this was laid a few years ago by Mark Bradley's group.^[^ ^148^ ^]^ The group identified biocompatible olefins (acrylic and methacrylic monomers), which are often strong Michael acceptors and thus can undergo side reactions. Furthermore, they have succeeded in generating free radicals in the cellular environment, enabling light‐mediated, olefin polymerization, while maintaining cell functions and viability. This makes it possible to alter, monitor, and govern cellular activity by generating polymers inside cells. The group could show that the intracellular polymers can influence cell movement or mark cells for extended tracking investigations if fluorescent polymers were used.

**Figure 14:**
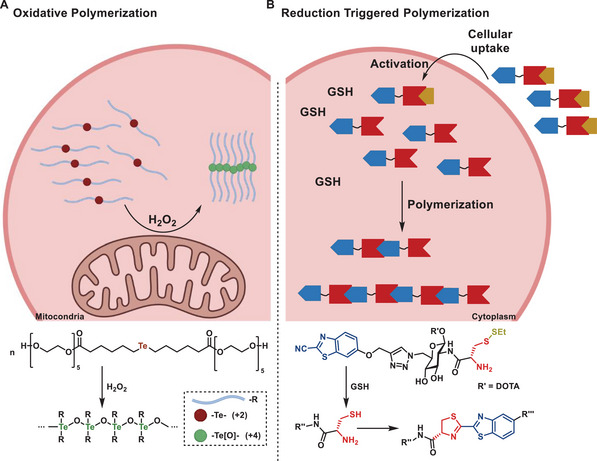
A) Oxidative polymerization of Te‐containing molecules by ROS;^[^ ^149^ ^]^ B) Condensation reaction between cyanobenzothiazole and cysteine upon reductive activation by GSH.^[^ ^150^ ^]^

A more biologically inspired application for polymerization reactions in living organisms has been demonstrated in recent years, where chain propagation was initiated either by oxidation or reduction. An oxidative polymerization in living cells was reported by Xu and coworkers in 2021 utilizing tellurium ethers undergoing ROS‐triggered polymerization.^[^ ^149^ ^]^ Organo‐tellurides in the oxidation state + 2 can form tetravalent Te‐O polymers under oxidation (**Figure** **14**A). Due to intrinsic differences in the oxidation stress of both healthy and cancer cells, this reaction could selectively take place in cancer cells and induce apoptosis via the self‐amplification mechanism. Exactly the opposite trigger was used by the Rao group.^[^ ^150^ ^]^ They have performed an in situ polymerization in mice (Figure 14B). A caged beta‐aminothiol (cysteine derivative) undergoes GSH‐mediated deprotection in the first step and, after this activation, a traceless polycondensation reaction with cyanobenzothiazole, forming the linear polymers at the tumor site.

Although great progress has been made in recent years to facilitate polymerization reactions in vitro and in vivo, much work still needs to be done to optimize the reaction conditions and ensure that the polymerization reactions do not have harmful effects on the cells.

#### 2.4.2 Depolymerization by Covalent Bond Breakage

**Figure 15:**
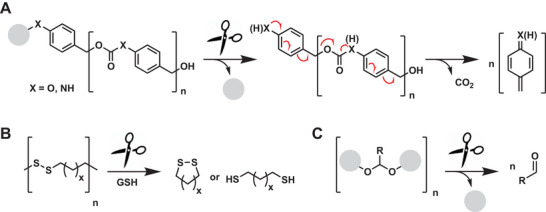
Schematic representation of self‐immolative polymers^[^ ^151^ ^]^ by A: 1,6‐elimination reaction, B: Disulfide reduction, or C: Acetal hydrolysis.

In clear contrast to the cascade reactions shown so far, self‐immolative polymers are another class of molecule, that utilizes the cleavage of several bonds as a reaction mechanism.^[^ ^140^, ^151^, ^152^ ^]^ Self‐immolative polymers are a class of macromolecules that can undergo spontaneous depolymerization or degradation triggered by an internal or external stimulus, leading to the release of small molecules or fragments. These materials have gained increasing attention in biomedical applications due to their potential as drug‐delivery vehicles, molecular probes, and biomaterials. These polymers can be designed to promote cell adhesion, proliferation, and differentiation, while also degrading in a controlled manner to facilitate tissue regeneration. Stimuli‐sensing trigger units used for these macromolecules can be UV‐ or NIR light, H_2_O_2_, or GSH.^[^ ^151^ ^]^ Backbone degradation can be caused by 1,4 or 1,6‐elimination of oligo carbamates or carbonates, releasing CO_2_ (**Figure** **15**A). The pKa value of corresponding phenols or anilines is curtailed, as release in living organisms cannot be achieved for pKa values that are too high.

Besides this “chain‐degradation” mechanism, there is also the possibility of a “block‐degradable” polymer. Here, the repeating unit of the backbone is, for example, an acetal or ketal, which is hydrolyzed in acidic cellular compartments, or a disulfide which is cleaved into a redox‐active section (Figure 15B,C).

Despite the potential advantages of self‐immolative polymers for in vivo application, there are still several challenges that need to be addressed. For example, the degradation products of these materials can be cytotoxic or immunogenic, so careful consideration must be given to the selection of monomers and linkages used in their synthesis. In addition, the degradation kinetics of self‐immolative polymers must be tuned to ensure that the material degrades at an appropriate rate for the desired application.

## 3 Monitoring and Characterization of Chemical Reactions In Vivo

**Figure 16:**
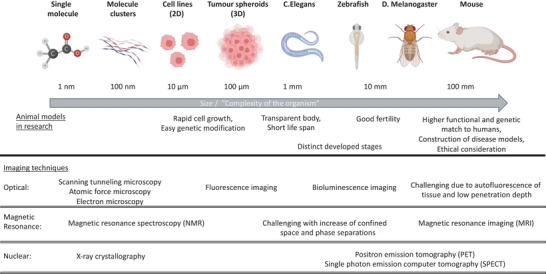
Overview of models of living organisms and suitable imaging techniques in research.

The analysis and characterization of chemical reactions as well as the (newly) synthesized products are crucial to optimize the underlying chemistry, to minimize side reactions, and receive a better understanding of the generated 3D structures. Currently, available imaging techniques for complex systems can be divided into three categories, namely optical, magnetic resonance, and nuclear methods (**Figure** **16**).^[^ ^153^ ^]^ This section discusses the strengths and limitations of established methods as well as more recent developments in preclinical imaging.

One of the emerging areas for analysis of complex systems is in microscopy, such as fluorescence, bioluminescence, and Correlative Light Electron Microscopy (CLEM) as noninvasive techniques for imaging orthotopic tumor models in mice. To date, the most frequently applied method for tracing complex processes is fluorescence spectroscopy.^[^ ^28^ ^]^ However, this technique only provides us with information on the interaction of two or more fluorescent molecules. Further, autofluorescence of tissue can limit its applicability in living organisms.^[^ ^154^ ^]^ As only light‐emitting molecules are detected, dyes need to be conjugated to the molecule of interest, which could affect the structure, dynamics, localization, interaction partners, and bioactivity of the target compound in the highly complex system. The challenge of considering the size/lipophilicity, charge, and metabolic stability of the fluorophore has been considerable.^[^ ^155^ ^]^

**Figure 17:**
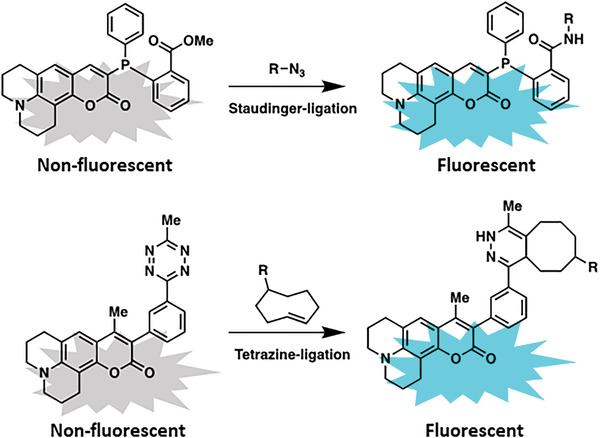
Selected examples of “turn‐on” fluorescents by Staudinger‐ligation (top) and tetrazine‐ligation (bottom).

Besides low molecular weight dyes, fluorescent proteins^[^ ^156^ ^]^ such as quantum dots have been used (e.g., green fluorescent protein) that can be expressed by cells or fluorescent nanoparticles that do not bleach over extended observation times.^[^ ^157^, ^158^ ^]^ To monitor chemical reactions, so‐called “turn‐on” fluorophores have been introduced (**Figure** **17**).^[^ ^159^, ^160^ ^]^ These molecules are “activatable probes”, which show fluorescence after a chemical reaction.^[^ ^157^ ^]^

Furthermore, it is possible to place a fluorescent dye and a fluorescent “quencher” next to each other and cleave the connecting linker by a chemical reaction. The donor fluorophore in its excited state is able to transfer its excitation energy to a neighboring acceptor fluorophore, resulting in the emission of the characteristic fluorescence of the acceptor.^[^ ^161^ ^]^ This so‐called Förster resonance energy transfer (FRET) can provide information on how close the two dyes are to each other and whether cleavage of the linker has occurred.^[^ ^162^ ^]^

This analytical tool, which has been used in cells or tissue, gives information about the proximity of two reagents or a successful conjugation reaction indicated by fluorescence probes. In vivo, non‐specific imaging agents cannot be washed away as easily as in cell experiments. Therefore, “turn‐on” probes, which accumulate mainly in target tissue, have been used to lower background noise. However, a major concern of almost all “turn‐on” fluorophores used in vitro and in vivo is that linker cleavage may occur by some off‐target stimuli or metabolization of the fluorophore, resulting in off‐target cleavage.

While optical microscopy can be used for imaging living systems, there are also some drawbacks and limitations to this technique, including limited penetration depth and phototoxicity.^[^ ^163^ ^]^ For example, confocal microscopy can only penetrate up to about 1 millimeter into tissue. This can be improved by applying two‐photon microscopy by several millimeters.^[^ ^164^, ^165^ ^]^ However, imaging structures and processes that are located deep within the tissue remain difficult. Living tissues can be damaged by the use of high‐intensity light sources known as phototoxicity. This can result in cell death, tissue damage, and other adverse effects.^[^ ^166^ ^]^ In addition, in vivo imaging by optical microscopy can be affected by motion artifacts, such as breathing or heartbeats. These artifacts can cause blurring or distortion of the images, which makes the interpretation of the data difficult.

**Figure 18:**
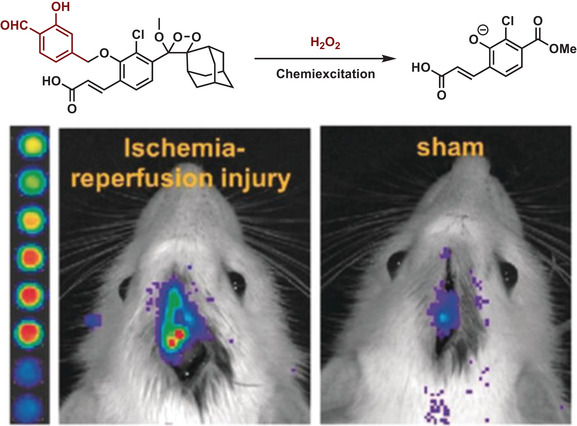
Real‐time monitoring of hydrogen peroxide in rat brains by combining sensing strategy and a peroxide bond excited chemiluminescent scaffold. Reproduced with permission.^[^ ^170^ ^]^ Copyright 2020, Wiley‐VCH.

Another optical method that does not require excitation by an external light source is bio‐ or chemiluminescence, which has been used for monitoring chemical reactions in vitro and in vivo.^[^ ^167^ ^]^ This imaging technique has been developed with *C. Elegans* and Zebrafish models due to their almost transparent bodies. Bioluminescence can be achieved by the enzymatic oxidation of luciferin (substrate) by luciferase (enzyme).^[^ ^168^, ^169^ ^]^ The intermediate of this oxidation is an unstable dioxetanone, which decomposes under light emission. Caged forms of luciferin can be activated by chemical triggers, allowing us to monitor the reaction process. Shabat and co‐workers have also designed a variety of 1,2 dioxetanes as chemoluminescent systems for in vivo imaging (**Figure** **18**).^[^ ^170^ ^]^

Nowadays, it is possible to determine the chemical properties (e.g., chemical composition, atomic arrangement, etc.) of “pure” chemical compounds as well as their inter‐ and intramolecular interactions (e.g., tertiary and quaternary structures of biomacromolecules). However, the analysis of the structure of (macro)molecules or chemical reactions in complex mixtures, for example, living systems, is very challenging due to the low analyte and product concentrations as well as the complex and dynamically changing environments.

Kurt Wüthrich was awarded the Nobel Prize in Chemistry in 2002 for applying nuclear magnetic resonance spectroscopy to determine the 3D structure of biopolymers in solution, with atomic resolution, which has become increasingly important in recent times.^[^ ^171^, ^172^, ^173^ ^]^ However, there is still no protocol available that allows us to follow chemical reactions in cells by applying NMR spectroscopy. Conformational changes of proteins caused by noncovalent interactions upon ligand binding, covalent post‐translational modifications, or protein–protein interactions have been the main focus of study in living cells. Improved signal‐to‐noise ratios have been observed after ^13^C or ^15^N enrichment in the expressed proteins, which has been crucial when connecting 3D structures to protein function in vivo by in‐cell NMR. With the development of new NMR hardware, new methods in sample preparation, and combination with other techniques, in‐cell NMR will play an increasingly important role in structural biology and drug discovery.^[^ ^174^ ^]^ A medical application of NMR is magnetic resonance imaging (MRI) enabling the visualization of 3D structures in complex living systems such as mice or humans, due to its high penetration depth.^[^ ^175^, ^176^, ^177^ ^]^ This non‐invasive imaging technique uses a strong magnetic field and radio waves to generate detailed images of the body. It can be used to visualize the internal structures of the body, including organs, soft tissue, and bones. It is particularly useful for diagnosing diseases and injuries in the brain, spinal cord, joints, and other internal organs. One of the advantages of this imaging method is that it does not use ionizing radiation, unlike X‐rays and computed tomography (CT) scans. This makes MRI safer for patients who need to undergo frequent imaging, such as those with cancer or chronic conditions. However, MRI does have some limitations and potential drawbacks, including expense, limited availability, and long scan times.

**Figure 19:**
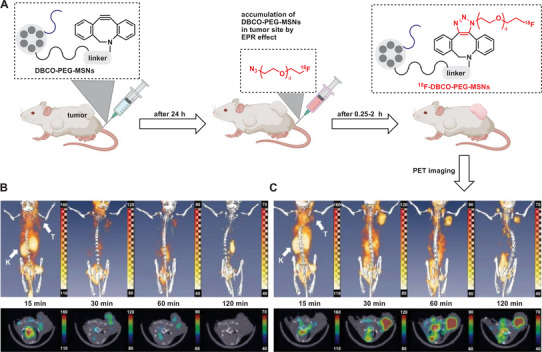
Pre‐targeting/labeling protocol for in vivo click reaction. B) 3D PET images (top) and transversal slides (bottom) of a U87 MG tumor‐bearing mouse injected with ω‐[18F]fluoro‐pentaethylene glycolic azide without pretargeting. C) 3D PET images (upper row) and transversal slides (lower row) of a U87 MG tumor‐bearing mouse injected with ω‐[18F]fluoro‐pentaethylene glycolic azide with pretargeting using DBCO‐PEG‐NPs. Reproduced with permission.^[^ ^181^ ^]^ Copyright 2013, Wiley‐VCH.

A nuclear, non‐invasive method suitable for monitoring chemical reactions is positron emission tomography (PET).^[^ ^178^ ^]^ This imaging method of nuclear medicine uses weakly radioactively labeled substances that are visualized at certain locations in the organism, often short‐lived positron emitters such as ^15^O, ^13^N, ^11^C, and ^18^F.^[^ ^179^, ^180^ ^]^ Since the 3D localization in the organism (even in deep tissue layers) is possible, it also allows us to monitor pre‐targeting chemical (click) reactions. Lee et al. described the use of in vivo strain‐promoted click chemistry to connect an [^18^F]‐radiotracer to mesoporous silica nanoparticles (**Figure** **19**).^[^ ^181^ ^]^ The prepared nanoparticles were injected into mice and accumulated in tumors via the EPR effect.^[^ ^182^ ^]^ After 24 h, the radiotracer was injected, and PET images were recorded. In this way, pre‐targeted mice showed a much higher ligand uptake into tumor tissue than the non‐targeted derivative, confirming that the copper‐free click reaction proceeds efficiently in vivo.

To date, a combination of approaches is often used, uniting the advantages of individual monitoring methods. An example is CLEM spectroscopy, which involves two main steps.^[^ ^183^, ^184^ ^]^ First, the sample is imaged using LM, which allows the identification and localization of specific structures or molecules of interest. Second, the same sample is imaged using EM, which provides high‐resolution structural information of the same area of the sample previously imaged by LM. This method has several advantages over traditional EM or LM alone. It allows for the visualization of a specific target in the context of its larger environment, providing more information about its function and role. CLEM can also be used to validate results obtained from LM, which typically has higher throughput and less sample preparation requirements than EM.

Another area of expanding research is the development of multimodal scanners, such as Computed Tomography‐Positron Emission Tomography (CT‐PET), to overcome individual limitations of a modality and to provide more details of tumors.^[^ ^185^ ^]^ The CT component of the imaging procedure provides the anatomical details of the body, while the PET component provides functional information on how body tissues are working at the cellular level. The combined images from CT and PET allow doctors to better identify the location and extent of abnormalities such as tumors, as well as monitor treatment response over time.

As chemists, we are required to perform analysis with single‐atom resolution. To date, translating this for in vitro and in vivo characterization is a challenging task due to the complexity and sensitivity of biological systems. However, there are some techniques that can provide atomic resolution insights into biological systems. Cryo‐electron microscopy (cryo‐EM) is a powerful technique that can be used to visualize biological molecules, such as proteins, at atomic resolution.^[^ ^186^, ^187^, ^188^, ^189^ ^]^ Cryo‐EM works by freezing samples in liquid nitrogen and imaging them using an electron microscope. Recent advances in cryo‐EM have made it possible to visualize large macromolecular complexes and even small molecules with sub‐angstrom resolution. Further, X‐ray crystallography can be used to determine the 3D structure of biological molecules.^[^ ^190^, ^191^, ^192^ ^]^ This is done by shining X‐rays through a crystal of the molecule and analyzing the diffraction pattern that is produced with atomic resolution. Another powerful technique for in vitro applications is scanning tunneling microscopy (STM), which uses a sharp tip to measure the electrical conductivity of individual atoms on a surface, as well as atomic force microscopy (AFM) which can also be used to image and manipulate individual atoms on a surface.^[^ ^193^, ^194^ ^]^ In both cases, the ability to manipulate and measure individual atoms will allow us to develop new materials and devices. All these extraordinary approaches have in common that they cannot be applied directly to the living system, but only on single (dead) cells, or on tissue slices.

## 4 Outlook

Despite the award of the Nobel Prize in Chemistry, research on the controlled formation of chemical reactions in living systems is still in its infancy. Click chemistry developed over the last two decades is a powerful tool for in vivo chemistry that involves the rapid and efficient formation of covalent bonds between two molecules. In the laboratory, click reactions are highly specific and selective and can be used to conjugate molecules for a wide range of applications. However, nature is more complicated than any laboratory condition or process and there are still various challenges that need to be overcome, such as optimizing the conjugation chemistry, achieving efficient and selective organelle targeting and local release, and ensuring the safety and efficacy of the resulting conjugates. Furthermore, the toolbox of chemical reactions that can be carried out on living systems is still quite primitive, to date only a few linkages are possible. The diversity of the nature of chemical bonds is far from being achievable with current ligation techniques.

Although there is still a long way to go, these methods could revolutionize our understanding of molecular processes in living systems. The potential insights and applications justify the effort. One could imagine that based on precisely controlled synthetic chemistry, structures could be assembled and disassembled in cells in a controlled manner to create functional organelles or to generate minimal cells, both involving complex reaction networks. The necessary foundations for these discoveries are currently being laid. Self‐immolative polymers hold significant potential for in vivo applications, but further research is needed to optimize their design and characterization for specific biomedical applications. Chemical triggers can be used to selectively cleave bonds in specific cellular compartments. For example, pH‐sensitive linkers can be used to release a drug or therapeutic agent only in a specific pH range. Similarly, redox‐active molecules can be used to selectively release a therapeutic agent in a specific redox environment. In vivo drug synthesis by bioconjugation has the potential to revolutionize drug development by creating highly specific and effective therapies with reduced side effects.

A current limitation is the lack of suitable analytical methods to elucidate structures and chemical reactions under dilute conditions with atomic resolution in complex biological environments. Exciting methods such as in‐cell NMR and quantum sensing are just emerging. Current theranostic methods often require invasive sampling of body fluids such as blood or spinal fluid. Advances in imaging techniques have significantly improved the choices available for in vivo analysis in animal research, particularly in the field of oncology. The development of existing technologies has led to major advances in the miniaturization of imaging techniques such as MRI, PET, and CT, enabling higher‐resolution visualization in smaller animals. Overall, these techniques provide researchers with powerful tools to study the structure and properties of biological systems on the atomic scale, which is essential for understanding biological processes and developing new therapeutics. However, these techniques also pose limitations and challenges in terms of sample preparation, imaging conditions, and data interpretation. To date, there is no method that allows us to obtain molecular information about the reaction and the products formed in the cells. Instead, reactions can be detected indirectly by generating a signal, such as fluorescence or bioluminescence, if the reactants are designed accordingly.

Nevertheless, great progress has been made in the development of chemical reactions in living systems in view of how new this field of research is in comparison to how much time nature has had to develop its unchallenged precision. If you review this field 50 years from now, there will certainly be unimaginable advancements made in investigating important biological questions and treating diseases.

## Conflict of Interest

The authors declare no conflict of interest.
